# Bioprocess exploitation of microaerobic auto-induction using the example of rhamnolipid biosynthesis in *Pseudomonas putida* KT2440

**DOI:** 10.1186/s13036-025-00478-z

**Published:** 2025-01-18

**Authors:** Jakob Grether, Holger Dittmann, Leon Willems, Tabea Schmiegelt, Elvio Henrique Benatto Perino, Philipp Hubel, Lars Lilge, Rudolf Hausmann

**Affiliations:** 1 Department of Bioprocess Engineering, Institute of Food Science and Biotechnology, University of Hohenheim, Fruwirthstr. 12, 70599 Stuttgart, Germany; 2 Core Facility Hohenheim, Mass Spectrometry Core Facility, University of Hohenheim, Ottilie-Zeller-Weg 2, 70599 Stuttgart, Germany

**Keywords:** *Pseudomonas putida*, Biosensor, Microaerobic, Auto-induction, Bioprocess design, Rhamnolipid, Bioreactor, Strain engineering

## Abstract

**Background:**

In biomanufacturing of surface-active agents, such as rhamnolipids, excessive foaming is a significant obstacle for the development of high-performing bioprocesses. The exploitation of the inherent tolerance of *Pseudomonas putida* KT2440, an obligate aerobic bacterium, to microaerobic conditions has received little attention so far. Here low-oxygen inducible promoters were characterized in biosensor strains and exploited for process control under reduction of foam formation by low aeration and stirring rates during biosynthesis of rhamnolipids.

**Results:**

In this study, homologous promoters of *P. putida* inducible under oxygen limitation were identified by non-targeted proteomic analyses and characterized by fluorometric methods. Proteomics indicated a remodeling of the respiratory chain and the regulation of stress-related proteins under oxygen limitation. Of the three promoters tested in fluorescent biosensor assays, the promoter of the oxygen-sensitive *cbb3-*type cytochrome c oxidase gene showed high oxygen-dependent controllability. It was used to control the gene expression of a heterologous di-rhamnolipid synthesis operon in an auto-inducing microaerobic two-phase bioprocess. By limiting the oxygen supply via low aeration and stirring rates, the bioprocess was clearly divided into a growth and a production phase, and sources of foam formation were reduced. Accordingly, rhamnolipid synthesis did not have to be controlled externally, as the oxygen-sensitive promoter was autonomously activated as soon as the oxygen level reached microaerobic conditions. A critical threshold of about 20% oxygen saturation was determined.

**Conclusions:**

Utilizing the inherent tolerance of *P. putida* to microaerobic conditions in combination with the application of homologous, low-oxygen inducible promoters is a novel and efficient strategy to control bioprocesses. Fermentation under microaerobic conditions enabled the induction of rhamnolipid production by low oxygen levels, while foam formation was limited by low aeration and stirring rates.

**Supplementary Information:**

The online version contains supplementary material available at 10.1186/s13036-025-00478-z.

## Background

Biomanufacturing microbial surfactants usually requires aerobic processes. However, maintaining high dissolved oxygen levels necessitates high aeration and stirring rates, which often promote strong foaming [1]. Foam in bioreactors impedes the process performance by, among other things, reducing the usable reactor volume, trapping biomass in foam lamellae and possibly leading to losses of biomass and product in piping due to overfoaming [2]. *Pseudomonas putida* KT2440 is a strictly aerobic bacterium that has proven to be a safe (HV1) [3] model bacterium and promising production strain for several compounds, such as bioplastics, muconic acid, 3-(3-hydroxyalkanoyloxy) alkanoic acid (HAA) or rhamnolipids [4–8]. *Pseudomonas putida* as a host for heterologous production of rhamnolipids has gained attention for its non-pathogenic character and robustness. The associated capability to withstand industrial-scale stress was shown recently [9], while a glucose-based industrial-scale rhamnolipid production has been announced [10]. Still, excessive foam formation of surface-active compounds is considered as a huge obstacle in the development of bioprocesses [2]. The strong foaming properties of rhamnolipids can lead to strong foam formation even at low product titers [11]. Such titers are reached early in bioprocesses, since usually, production strains with constitutive expression system are used [12–14]. Different strategies have been investigated to reduce foam formation in processes for the production of surface-active bioproducts, such as the excessive use of antifoaming agents [15], implementation of bubble-free gassing using a membrane aerator [16], head space aeration in combination with high pressure [7] or in situ liquid-liquid extraction in a bubble column reactor [17]. Despite the innovative work that has been undertaken to face the obstacles of excessive foam formation, the approaches suffer from complexity, low controllability or scalability issues. Separation of growth and production phases in bioprocesses by a control of oxygen supply has been discussed for facultative anaerobic bacteria [18]. In the industrial workhorse *Bacillus subtilis*, oxygen availability has been used as a trigger for production of the biosurfactant surfactin by the use of anaerobiosis-induced promoters of nitrate and nitrite reductase [19]. However, using the genetically regulated inherent tolerance of *P. putida* KT2440 to microaerobic conditions has not yet been exploited as a chance for bioprocess control.

In this study, proteome analysis was performed for detecting the adaption of *P. putida* to a microaerobic environment, allowing the indirect identification of promoters inducible under oxygen-limited conditions. Subsequently, selected target promoters were utilized to develop *P. putida* fluorescent biosensor strains. Overall, the sensor strain using the promoter region of the *cbb3*-type cytochrome c oxidase operon showed the most promising controllability depending on levels of dissolved oxygen and was therefore applied in dual-phase microaerobic bioprocesses. In this way, low oxygen content-inducible heterologous di-rhamnolipid production could be established with a clear separation of growth and production phases by keeping aeration and agitation rates low, resulting in a reduction of excessive foam formation. Finally, the implemented expression system was used to establish a bioprocess allowing an auto-inducible gene expression in an oxygen-dependent manner for facilitated process control.

## Methods

### Cultivation condition

As standard cultivation method in shake flask, cultures were grown using a flask filling volume of 10% at 30 °C with an agitation speed of 120 rpm. For analysis of the fluorescent biosensor signals to decreased oxygen availability in shake flask, agitation was decreased from 120 rpm to 40 rpm, when OD_600_ = 1 was reached. To compare the rhamnolipid production with *P. putida* pJG-*cco1::rhlCAB* in shake flasks, the non-induced cultures were agitated at 120 rpm, while the induced cultures were agitated at 60 rpm. Data and error bars represent average values and standard deviation of 3 biological triplicates.

For all experiments, the following seed train was used: 10 mL of LB medium (10 g/L peptone, 5 g/L yeast extract, 5 g/L NaCl) was inoculated with 10 µL of a glycerol stock of a selected strain. The pre-culture was cultivated for 24 h before being used for inoculation in SupM medium [15] consisting of 4.4 g/L Na_2_HPO_4_ × 2 H_2_O, 1.5 g/L KH_2_PO_4_, 1 g/L NH_4_Cl, 0.2 g/L MgSO_4_ × 7 H_2_O, 0.02 g/L CaCl_2_ × 2 H_2_O, 0.006 g/L FeCl_3_, 30 g/L glucose, 10 g/L yeast extract and 1 mL/L of SL6 trace element solution. After 15 h of cultivation, the second pre-culture was used to inoculate the main culture, using ModR medium [15] consisting of 22 g/L KH_2_PO_4_, 2.6 g/L (NH_4_)_2_HPO_4_, 1.4 g/L MgSO_4_ × 7 H_2_O, 0.87 g/L citric acid, 0.01 g/L FeSO_4_ × 7 H_2_O, 5 g/L glucose, 10 mL/L of SL6 trace element solution (pH 6.8). SL6 trace element solution consisted of 0.3 g/L H_3_BO_3_, 0.2 g/L CoCl_2_ × 6 H_2_O, 0.1 g/L ZnSO_4_ × 7 H_2_O, 0.03 g/L MnCl_2_ × 4 H_2_O, 0.01 g/L CuCl_2_ × 2 H_2_O, 0.03 g/L Na_2_MoO_4_ × 2 H_2_O, 0.02 g/L NiCl_2_ × 6 H_2_O. All media contained tetracycline as selection marker (20 µg/mL). All chemicals were obtained from Carl Roth^®^, Germany.

### Determination of cell dry weight (CDW) conversion factor

To calculate the CDW from optical density at a wavelength of 600 nm (OD_600_), a conversion factor k = 3.5 L/g was determined. For this, *P. putida* strain *pSynPro8oT_rhlAB* was cultured in triplicates in ModR medium as described above. After measuring the OD_600_, samples of 10 mL were transferred in pre-dried (105 °C, 24 h) and pre-weighed 15 mL centrifuge tube, pelleted by centrifugation (15 min, 4.700 x *g*, 4 °C) and washed twice using 10 mL of 0.9% (w/v) NaCl by resuspension and centrifugation at the same parameters. The cell pellet was then dried (105 °C, 24 h) and weighed to determine the conversion factor k.

### Shake flask cultivation in N_2_-enriched atmosphere

Similar to the study of Hoffmann et al. [20], reduction of oxygen availability was achieved by flushing the flasks with N_2_. For shake flask cultivations with N_2_-enriched atmosphere, main cultures were grown in shake flasks (absolute volume of 500 mL) equipped with a tube design that allowed N_2_ supply and air output for atmosphere exchange in the headspace of the flasks (Supplementary Figure S1). In addition, a cannula was guided through the cellulose plug of the shake flask, allowing sampling with syringes. Cultures were grown under standard conditions, as described above, until an OD_600_ of 1 was reached. To displace most of the air in the headspace of the shake flasks, nitrogen was introduced for 30 s with a flow rate of 6 L/min, and cellulose plugs were sealed with airtight foil. Subsequently, cultures were continued to shake at 120 rpm, allowing limited cell growth relying on residual oxygen dissolved in the medium and left-over oxygen in the head space. During sampling via the syringe, nitrogen was supplied to maintain the N_2_-enriched atmosphere.

### Sampling for proteome analysis

A sample of 5 mL was taken from the shake flask and centrifuged for 5 min at 30 °C and 4.700 x *g*. The bacterial pellet was resuspended in 1 mL of pre-heated (90 °C) lysis buffer (4% (w/v) SDS, 100 mM Tris HCl, pH 8.5), then boiled at 95 °C for 5 min, and subsequently frozen at -80 °C until further analysis.

### LC-MS/MS proteomics sample preparation

Lysates were cleared by centrifugation at 20.000 x *g*, reduced and alkylated in 10 mM tris(2-carboxyethyl)phosphine HCl, 40 mM chloroacetamide for 20 min at 60 °C in the dark and precipitated for 16 h at -20 °C in 80% acetone. Precipitated proteins were pelleted by centrifugation at 20.000 x *g* for 15 min at 3 °C. Air dried protein pellets were solubilized in 0.2% sodium deoxycholate, 100 mM Tris HCl (pH 8.5) and protein concentrations were determined by a Bradford assay (Roti-Quant, Roth). Lysates were adjusted to a final protein concentration of 1 µg/µL. Thirty microliters of the lysate were used for protein extraction by Single-Pot Solid-Phase-enhanced Sample Preparation (SP3; 1:1 Mixture of SpeedBeads™ magnetic carboxylate modified particles 50 mg/mL; Cytiva; CAT No: 45152105050250 and 65152105050250) [21]. Proteins were bound to the magnetic beads by adding ethanol to a final concentration of 70%. Bead bound proteins were washed twice with 70% ethanol and digested on the beads in 0.2% SDC, 50 mM ammonium bicarbonat, in a protease to protein ratio of 1:100 trypsin (Roche) and 1:200 LysC (Walko) respectively (20 h, 37 °C, 800 rpm). Formic acid (FA) was added to the samples to a final concentration of 2%, precipitated SDC was pelleted by centrifugation at 20.000 x *g* for 15 min, peptides in the supernatant were concentrated and desalted on C18 Stage Tips as described by Rappsilber et al. [22] and dried under vacuum. Dried samples were dissolved in 0.1% TFA.

### LC-MS/MS proteome analysis

NanoLC-MS/MS experiments were performed on an Ultimate 3000 nano-RSLC (Thermo Fisher Scientific, Germany) coupled to an Exploris 480 mass spectrometer (Thermo Fisher Scientific) using a Nanospray-Flex ion source (Thermo Fisher Scientific). Peptides were concentrated and desalted on a trap column (µPAC Trapping column nano, PharmaFluidics, Belgium) and separated on a 50 cm µPAC nanoLC column (PharmaFluidics, Belgium) operated at constant temperature of 40 °C. Peptides were separated using a gradient with the following profile: 2% (700 nL/min) − 8% (300 nL/min) solvent B in 3 min, 8 − 20% (300 nL/min) solvent B in 27 min, 20 − 35% (300 nL/min) solvent B in 8 min, 35 − 95% (300 nL/min) solvent B in 3 min, isocratic 95% (300 nL/min) solvent B for 3 min, 95% (300 nL/min) − 2% (700 nL/min) solvent B in 5 minutes and isocratic 2% (700 nL/min) solvent B for 10 min. The solvents used were 0.1% FA (solvent A) and 0.1% FA in ACN/H_2_O (80/20, *v/v*, solvent B). MS spectra (m/z = 300 − 1.500) were detected in the Orbitrap at a resolution of 60.000 (m/z = 200). The maximum injection time (MIT) for MS spectra was set to 50 ms, the automatic gain control (AGC) value was set to 3 × 10^6^. Internal calibration of the Orbitrap analyzer was performed using lock-mass ions from ambient air as described in Olsen et al. [23]. The MS was operating in the data-dependent mode selecting the top 25 highest abundant peptide precursor signals for fragmentation (HCD, normalized collision energy of 30). For MS/MS analysis only precursor charge states from 2 to 5 were considered, the monoisotopic precursor selection was set to peptides, and the minimum intensity threshold was set to 1 × 10^5^. MS/MS scans were performed in the Orbitrap with a resolution of 150.000, isolation width was set to 1.6 Da. The AGC target was set to 7 × 10^4^, a max injection time was set to 40 ms and the first mass was set to 120 m/z. Dynamic exclusion was set to 60 s with a tolerance of 10 ppm.

### Proteomics MS data analysis

Raw files were imported into MaxQuant [24] version 1.6.2.10 for protein identification and label-free quantification (LFQ) of proteins. Protein identification in MaxQuant was performed using the database search engine Andromeda [25]. MS spectra and MS/MS spectra were searched against *P. putida* KT2440 sequence database and five additional sequences (UniProt ID: A0A6G8J3R6, Q51559, D2EDM4, Q8VLR2, Q7BTS0) downloaded from UniProt [26]. Reversed sequences as decoy database and common contaminant sequences were added automatically by MaxQuant. Mass tolerances of 4.5 ppm (parts per million) for MS spectra and 20 ppm for MS/MS spectra were used. Trypsin was specified as enzyme and two missed cleavages were allowed. Carbamidomethylation of cysteines was set as a fixed modification and protein N-terminal acetylation and oxidation were allowed as variable modifications. The ‘match between runs’ feature of MaxQuant was enabled with a match time window of 0.7 min and an alignment time window of 20 min. Peptide false discovery rate (FDR) and protein FDR thresholds were set to 0.01.

MaxQuant output file (protein groups table) was loaded into Perseus version 1.6.14.0 [27]. LFQ values from MaxQuant were log_2_-transformed and matches to contaminant (e.g., keratins, trypsin) and reverse databases identified by MaxQuant were excluded from further analysis. Significant changes in protein abundance between nitrogen treated cells and the control condition were analyzed by a Welch’s t-test (two sided, S0 = 0) and corrected for multiple hypothesis testing using permutation-based FDR statistics (FDR = 0.05, 250 permutations). Significant candidates were further evaluated on the basis of their log2-transformed LFQ fold changes as stated in the results section using a p-value cut-off of 0.05. For determination up- and downregulated proteins a cut-off of log_2 _(fold change) of 1 and − 1, respectively, was used.

### Microscopy

For determination of the cell length during different agitation speed in shake flasks, a volume of 10 µL bacterial culture was taken during the mid-growth phases and distributed on a glass slide. After air-drying for 2 min, bacteria were heat-fixed using a Bunsen burner, and 20 µL of 0.9% (w/v) NaCl solution were dropped on the sample prior to applying a cover slip. Cells were examined with a Zeiss Leica DM 2000 LED (Oberkochen, Germany) in phase contrast 3 with a 100x objective and photographs were taken with the Leica MC 170 HD (Wetzlar, Germany). Cell lengths were measured using ImageJ (version 1.54).

### Cloning procedure

Isolation of plasmid DNA and chromosomal DNA were done with innuPREP Plasmid Mini Kit 2.0 and innuPREP Bacteria DNA Kit, respectively (IST Innuscreen GmbH, Berlin, Germany), according to the manufacturer´s protocol. Primers were obtained from Eurofins (Ebersberg, Germany) and Thermo Fisher (Sindelfingen, Germany). For polymerase chain reactions (PCRs), Q5 high-fidelity polymerase and Taq-polymerase were used. All PCR kits were obtained from New England Biolabs (Frankfurt a. M., Germany). PCRs were conducted according to standard protocols of the manufacturer. Purification of PCR-based cloning DNA fragments was performed with the Monarch^®^ DNA Gel Extraction Kit (New England Biolabs, Frankfurt a. M., Germany).

All plasmids were cloned using Gibson Assembly [28]. For in-house prepared Gibson Assembly Master Mix (GAM), 320 µL of 5x ISO buffer (0.5 M Tris-HCl pH 7.5, 0.05 M MgCl_2_, 0.001 M of each deoxynucleotide (dATP, dGTP, dTTP, dCTP), 0.05 M dithiothreitol, 0.005 M NAD, 4 M PEG-8000) was mixed with 0.64 µL of 10 U/µL T5 exonuclease, 20 µL of 2 U/µL Phusion polymerase, 160 µL of 40 U/µL Taq ligase and water to a final volume of 1.2 mL. Aliquots of 15 µL were stored in -20 °C. For Gibson Assembly, an aliquot of GAM was mixed with a total of 5 µL of overhang compatible DNA fragments, and incubated for 1 h at 50 °C. Subsequently, the reaction mixture was directly used for transformation using electro-competent *P. putida* KT2440 or stored in -20 °C until further use.

For preparation of electro-competent *P. putida* strains, 10 mL of LB medium was inoculated with 10 µL of cryo-conserved bacteria (50% (v/v) glycerol) and incubated overnight. Next day, the cells were separated by centrifugation (5 min at 6.000 x *g*, room temperature) and washed twice in 0.3 M sucrose solution before the cells were resuspended in 2 mL sucrose solution. Competent cells were stored on ice until further use. For electroporation, 100 µL of competent cells were mixed with 1.5 µL of Gibson Assembly reaction mixture, mixed carefully, and then transferred to pre-cooled (-20 °C) electroporation cuvettes (1 mm gap) (Carl Roth, Karlsruhe, Germany). Subsequently, a voltage of 1.6 kV was applied for electroporation. The cells were resuspended in 1.5 mL of LB medium, and allowed to reconstitute for 2 h at 150 rpm and 30 °C. Afterwards, cells were plated on LB agar containing tetracycline (20 µg/mL).

For the construction of the plasmid pJG-*rhlAB*, a small non-functional region of 111 bp (54 bp downstream of the Rep protein coding sequence) was removed from the parental plasmid *pSynPro8oT_rhlAB*, allowing heterologous mono-rhamnolipid production in *P. putida*. To generate pJG-*EDR*, which served as a parental plasmid for cloning of the biosensor constructs, the plasmid backbone of pJG-*rhlAB* was fused with a *mGFPmut3*-coding region without any upstream located promoter region from the plasmid pEB1-*mGFPmut3* [29] and a *mAmetrine*-coding region from the plasmid pBad-*mAmetrine* [30]. Candidate promoter regions of *P. putida* KT2440 were amplified for subsequent transcriptional fusion with the *mGFPmut3*-gene. For this purpose, the intergenic regions upstream of the respective target genes were applied. In detail, for the biosensor plasmid pJG-*cco1::gfp*, the 224 bp upstream region of the gene *ccoN-1* (PP_4250) was amplified. For pJG-*adhP::gfp*, the 435 bp upstream region of the *adhP* (PP_3839) gene was amplified. The 510 bp upstream region of the gene *rmf* (PP_5502) was amplified for the construction of the biosensor plasmid pJG-*rmf::gfp*. For other purposes that are not associated with this manuscript, promoter regions for subsequent transcriptional fusion with the *mAmetrine*-gene were amplified. For higher efficiency during Gibson Assembly, a promoter of *mGFPmut3* was fused with a promoter of *mAmetrine* using overlap extension PCR, using compatible overlaps incorporating the bidirectional *tonB*-terminator sequence in between. The constructed promoter fusions were then combined with the compatible backbone of pJG-*EDR* via Gibson Assembly to generate the biosensor plasmids. A schematic visualization of the cloning procedure is provided in the supplementary data (Supplementary Figure S2).

For cloning the low-oxygen inducible di-rhamnolipid production plasmid pJG-*cco::rhlCAB*, the plasmid backbone of pJG-*rhlAB*, the promoter region of the *cco1*-operon from *P. putida* KT2440, and the *rhlC* gene from *P. aeruginosa* strain DSM 19880 were amplified and used for Gibson Assembly.

All cloned constructs were verified by Sanger sequencing (Eurofins Genomics GmbH, Ebersberg, Germany). The primers used for plasmid construction are provided in the supplementary data (Supplementary Table S1).

### Bioreactor cultivation

A pre-culture grown in LB medium was used to inoculate SupM-medium as a second pre-culture. Subsequently, exponentially growing cells were used for inoculation of the main cultures in ModR medium at an optical density (OD_600_) of 0.3. All cultivations were performed at 30 °C. The pO_2_ probe was calibrated using air or N_2_ with a mass flow of 0.133 vvm for 100% or 0% pO_2_ calibration, respectively.

The following bioreactor systems were used for different bioprocesses: For batch cultivations of the fluorescent biosensor strains, a 2 L bioreactor system (Labfors 4, Infors HT, Bottmingen, Switzerland) with 1 L working volume was used. For batch cultivation of the di-rhamnolipid producing strain, a 10 L bioreactor system (BIOSTAT^®^ B plus, Sartorius, Göttingen, Germany) with 6 L working volume was used. For fed-batch cultivation of the di-rhamnolipid producing strain, a 30 L bioreactor system (ZETA GmbH, Graz/Lieboch, Austria) with 15 L initial working volume was used. Feeding solution consisted of 400 g/L glucose, 22 g/L KH_2_PO_4_, 3.43 g/L KH_2_PO_4_, 0.01 g/L FeSO_4_ × 7 H_2_O, 0.1 mg/L CuCl_2_ × 2 H_2_O and 20 mg/L tetracycline. The pH was controlled at 6.8 with 1 M H_2_SO_4_ or 19% (w/v) NH_4_OH. If not otherwise stated, the air flow during fermentation was kept constant at 0.133 vvm. Oxygen levels were controlled via the stirrer, which was equipped with 2 (Infors and BIOSTAT bioreactors) or 3 (ZETA bioreactor) Rushton impellers. If necessary, antifoaming agent (SB590, Schill + Seilacher Struktol GmbH, Hamburg, Germany) was added manually.

### Fluorescence measurement

Fluorescence was measured using the FLUOstar Omega plate reader (BMG Labtech, Ortenberg, Germany). Therefore, 100 µL of a sample was pipetted per well into a flat bottom 96-well plate (TPP, Trasadingen, Switzerland) in technical triplicates. Fluorescence and OD_600_ were measured (bottom measurement) after shaking for 30 s at 300 rpm. The signal of mGFPmut3 was measured at 485/520 nm (ex./em.), gain was set to 1000. The fluorescence signal was divided by the OD_600_ measured by the plate reader to obtain the specific fluorescence (RFU/OD).

### Rhamnolipid extraction and measurement

Extraction and quantification of rhamnolipid was performed as described before [33]. In brief, culture was harvested and centrifuged (4 °C, 15 min, 4.700 x *g*). Two milliliters of cell-free culture supernatant were acidified 1:100 (*v/v*) with 85% *ortho-*phosphoric acid and extracted two times with 1.25:1 (*v/v*) ethyl acetate. Extracts were pooled and evaporated at 40 °C for 40 min at 10 mbar using a vacuum concentrator (RVC 2–25 Cdplus, Martin Christ Gefriertrocknungsanlagen GmbH, Osterode am Harz, Germany). For quantification, evaporated extracts were dissolved in acetonitrile and derivatized in a shaking incubator (ThermoMixer C, Eppendorf, Wesseling-Berzdorf, Germany) using equal volumes of 135 mM bromophenacyl bromide and 65 mM tri-ethyl-ammonium/-amin for 90 min at 60 °C and a shaking frequency of 1.400 rpm. Di-rhamnolipid (99% purity), obtained as a gift from former Hoechst AG (Frankfurt, Germany), was used as standard. Samples were applied on silica gel 60 HPTLC plates with fluorescence marker F^254^ (Merck, Darmstadt, Germany). Plates were developed in 30:5:2.5:1 isopropyl acetate: ethanol: water: acetic acid. For rhamnolipid quantification, absorption at 263 nm wavelength, mediated by bromophenacyl-groups of the derivatized rhamnolipid congeners, was measured.

### Analysis of foam formation in different aeration and stirring rates

Foaming was compared for different combinations of aeration and stirring rates. For this, the BIOSTAT^®^ B plus system was filled with 6 L of ModR medium containing 5 g/L of glucose, 25 µL/L antifoaming agent and 1.7 g/L of rhamnolipid, which was a gift from EVONIK Industries (Hanau, Germany). The system was aerated at 0 vvm, 0.133 vvm, 0.25 vvm and 0.5 vvm and stirred with different rates ranging from 100 to 900 rpm. For comparison, the foam height was measured (in cm) for each combination and the percentage of the headspace filled with foam was calculated.

## Results

### Adaptation of the *P. putida* proteome after reduction of oxygen availability

**Fig. 1:**
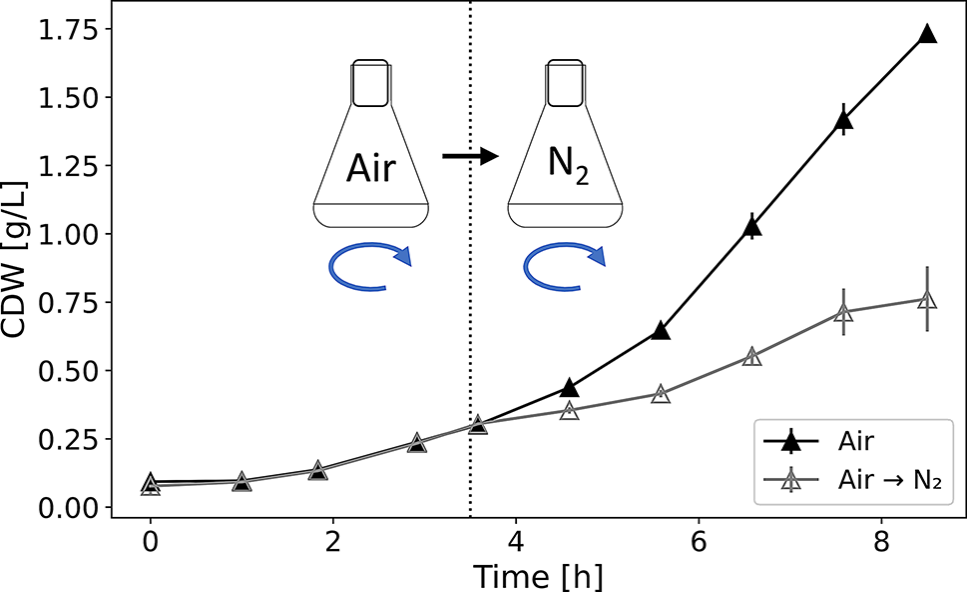
Time course of cell dry weight (CDW) in shake flask cultivations grown under ambient air (filled triangles) or shifted to a nitrogen-enriched atmosphere (empty triangles). Dotted line indicates the gassing of the flask headspace with nitrogen gas (N_2_)

To identify promoter candidates for the subsequent construction of low-oxygen inducible biosensor strains, the changes in the *P. putida* proteome after rapid reduction of oxygen availability were investigated. For this purpose, shake flask cultures were allowed to grow under continuous agitation (120 rpm), while the headspace atmosphere of the flask was exchanged with gaseous nitrogen (N_2_) in the early exponential growth phase (OD_600_ ≈ 1). As a result, a decreased culture growth, presumably due to lowered oxygen availability, was observed under these conditions (Fig. 1).

**Fig. 2:**
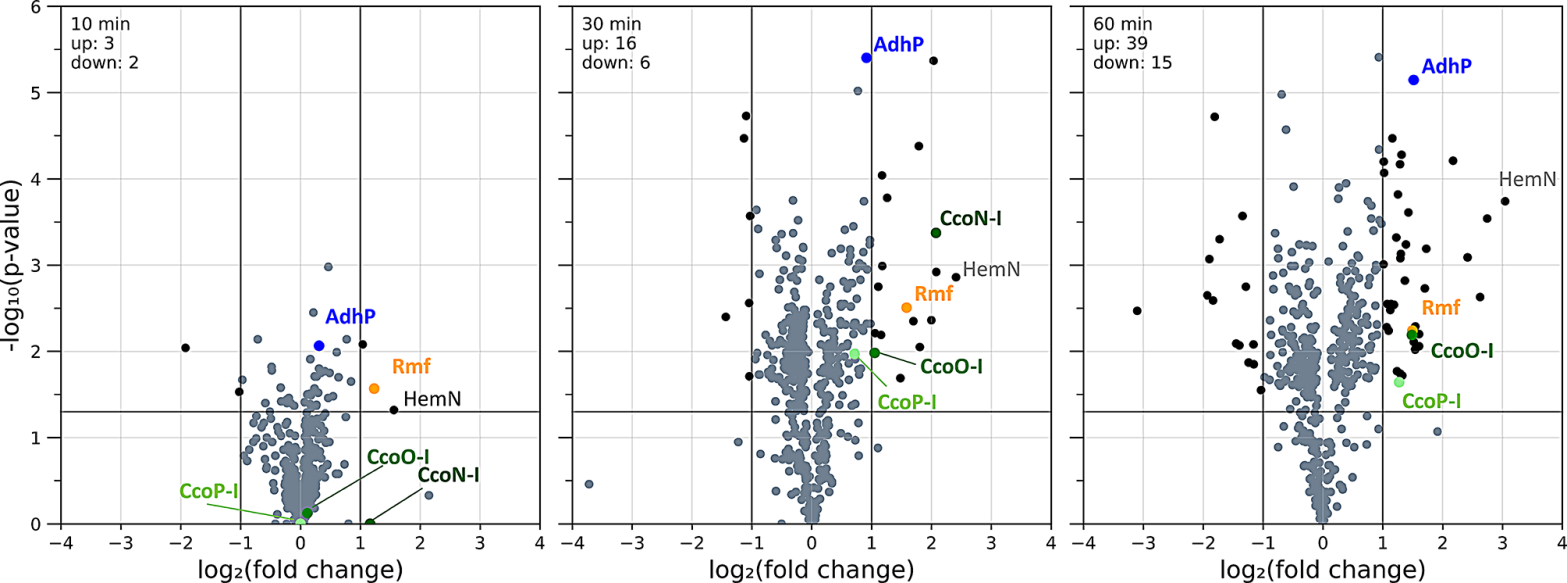
Volcano plots of *P. putida* proteome adaptations during exposure to an oxygen-depleted atmosphere after 10 (left), 30 (middle) and 60 min (right) of N_2_-mediated oxygen reduction. Vertical lines indicate doubled or halved protein abundances, as represented by the log_2_(fold change). Horizontal lines represent a significance level of 5%, as represented by a -log_10_(p-value) of 1.301

Samples for untargeted proteome analysis were taken after 10, 30 and 60 min of oxygen reduction and were compared to reference cultures grown under continuous agitation without atmosphere exchange. In proteome analysis, only proteins were considered that appeared significantly (*p* < 0.05) over- or underrepresented with respect to the control group in at least one sampling time point of the experimental time series (450 proteins in sum). After 10 min, only few significant changes were observed (43 significant protein changes, meaning 9.6% of all proteins). A dominant proteome adaptation could be detected after 30 min with 313 proteins (69.6% of all proteins) significantly changed in their abundance. After 60 min of growth in oxygen depleted atmosphere, the abundance of 335 proteins was significantly changed, resembling 74.4% of all proteins. However, significant fold changes in protein abundances were generally modest, as most of the proteins showed less than a two-fold change (Fig. 2).

Especially proteins functionally related to the respiratory chain and stress response were observed overrepresented in oxygen-depleted cultivation conditions. For instance, a highly increased protein abundance was found for the *cbb3*-type cytochrome c oxidase operon 1 (a tetracistronic operon composed of the genes *ccoN*, *ccoO*, *ccoQ* and *ccoP*, here referred to as *cco1*-operon). This protein complex has a high affinity to oxygen and is known to be expressed only under microaerobic conditions [32]. In addition, proteins involved in the oxidative stress response, such as the catalase KatA and Azurin were found in increased abundance over time. An alcohol dehydrogenase (encoded by the gene *adhP*) was among the proteins with strongest increase in abundance, which is in accordance with the proteomic changes of *P. putida* KT2440 showing after long-term (7.6 h) oxygen oscillation conditions described in [33]. The ribosome modulation factor Rmf, which is expressed in other bacteria, such as *E. coli* during stationary phase and low growth rates [34], was also among the first strongly overrepresented proteins. The full dataset of the proteome analysis is provided in the supplementary material (Supplementary Table S2).

### Construction of hypoxia biosensors

**Fig. 3:**
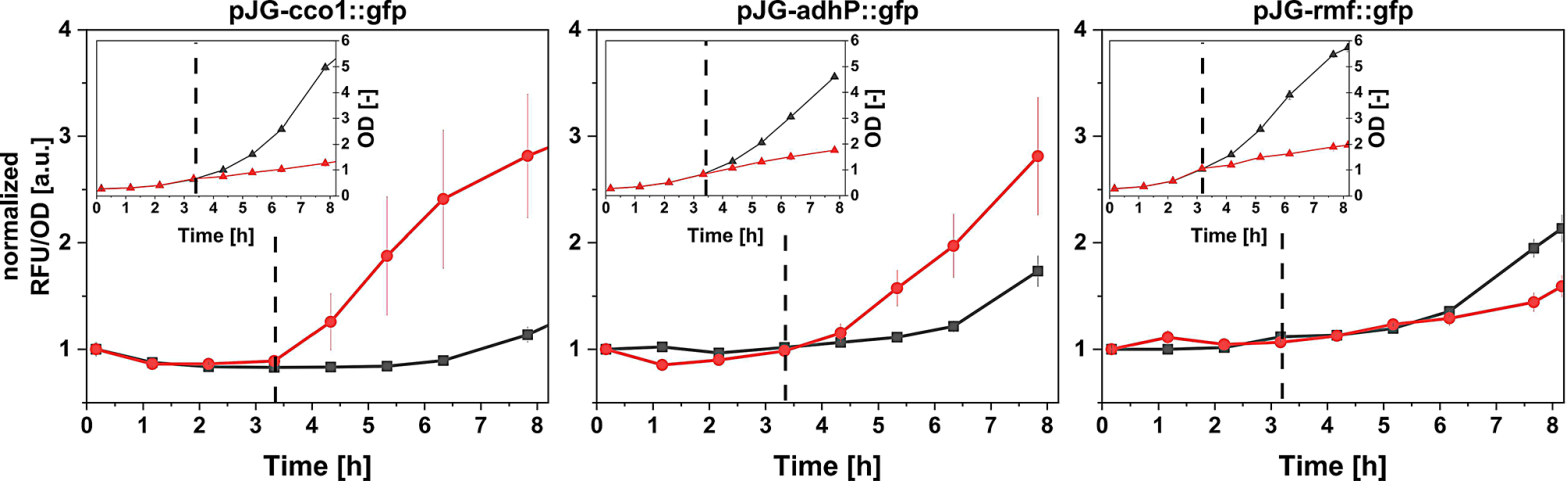
Time course of specific fluorescence (RFU/OD) and optical density (OD) of *P. putida* biosensor strains under reduced shake flask agitation. Main graphs: Specific fluorescence (RFU/OD) of stressed (red dots) and control cultures (black squares). Inset graphs: Optical density (OD) of stressed (red triangles) and control (black triangles) cultures. The dashed lines indicate the reduction of agitation from 120 to 40 rpm

To find potential promoter candidates that can be used for bioprocess control via oxygen availability, fluorescent biosensor strains were constructed and tested for their functionality. For this purpose, based on the proteome data and the available literature, the upstream regions of the *cco1*, *adhP* and *rmf*, respectively, were transcriptionally fused with the *mGFPmut3*-gene (encoding a fast maturing GFP derivative [35]) and cloned into a derivative of *pSynPro8oT_rhlAB* [13]. Accordingly, the constructed biosensor plasmids are referred to as pJG-*cco1::gfp*, pJG-*adhP::gfp* and pJG-*rmf::gfp*. To evaluate the functionality of the constructed biosensor strains, shake flask cultivations with decreasing agitation were conducted. Agitation reduction led to a decrease of oxygen input into the culture and, as a result, an induction of the *mGFPmut3* gene expression. The specific fluorescence (fluorescence per backscattered light, RFU/OD) of *P. putida* pJG-*cco1::gfp* and pJG-*adhP::gfp* increased strongly with respect to the control culture, which was constantly cultivated at high agitation (Fig. 3). Specifically, the increase in fluorescence was rapid especially for pJG-*cco1::gfp*, with fold changes of approximately 1.5, 2 and 3.5 after one, two and three hours post induction, respectively. For pJG-*adhP::gfp*, these magnitudes of fold changes in fluorescence were reached with a delay of 1 h. After 4.5 h post induction, similar fluorescence fold changes of ≈ 2.8 were observed for both strains. In contrast, *P. putida* pJG-*rmf::gfp* did not show an increased fluorescence after decreasing the agitation, indicating that oxygen limitation by reducing the shaking velocity is not sufficient to induce the *rmf*-promoter. Instead, the fast-growing control culture showed increased fluorescence at late growth stages, which indicated the transition to the stationary phase (Supplementary Figure S3). Overall, since the *P. putida* sensor strain pJG-*cco1::gfp* showed the strongest and most immediate induction in reduced agitation, further applications and characterizations were done with this strain.

### Characterization of the *cco1-*promoter activity

**Fig. 4:**
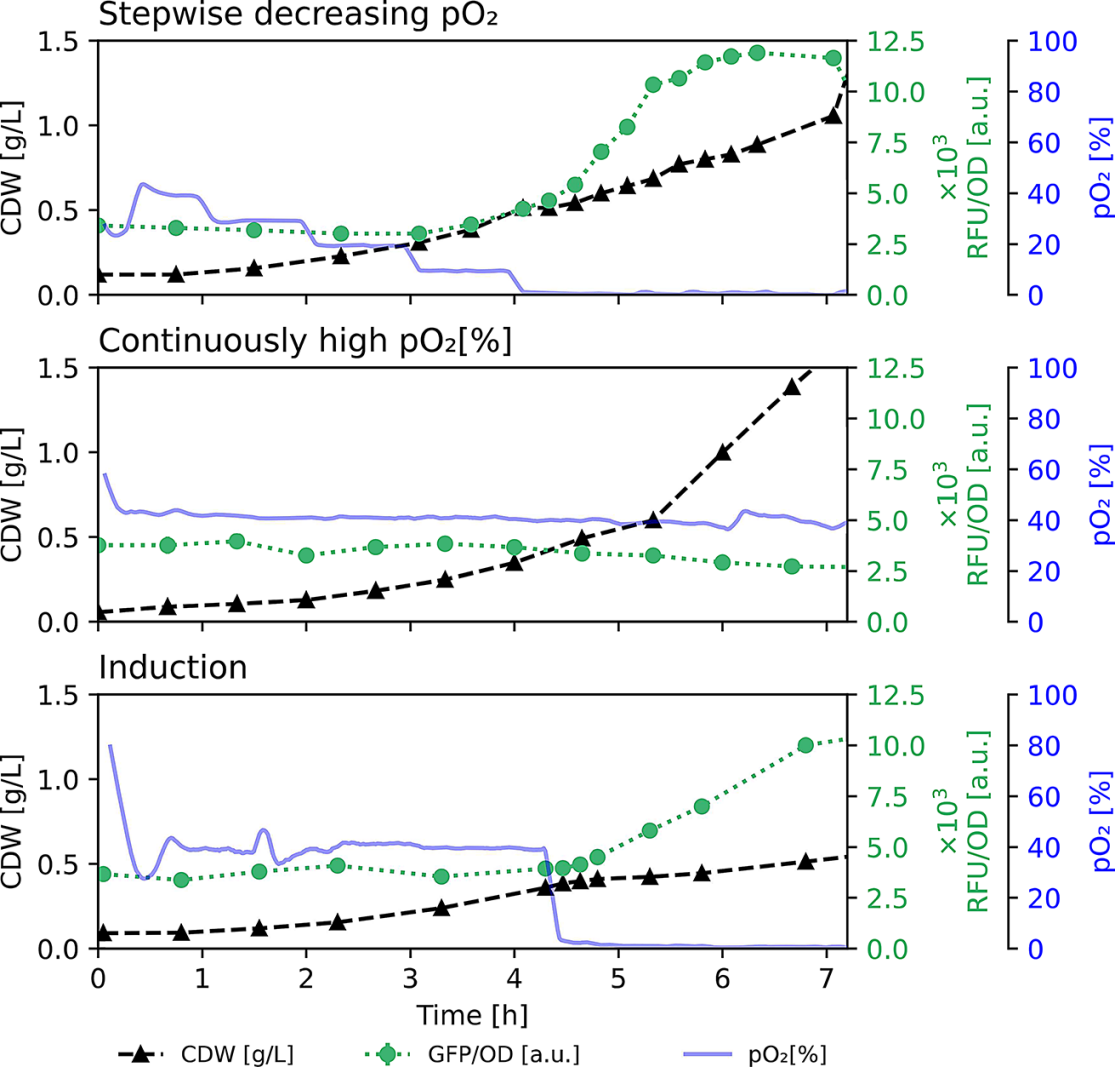
Time course of the *P. putida* pJG-*cco1::gfp* biosensor activity during different oxygen availability profiles. Growth (CDW, black triangles) and specific fluorescence (RFU/OD, green dots) of *P. putida* pJG-*cco1::gfp*, oxygen levels (pO_2_, blue line). **Top panel**: Stepwise decrease from 40–0%. **Mid panel**: Constant pO_2_ of 40%. **Bottom panel**: Drop of pO_2_ from 40–0%

To figure out, at which oxygen saturation level the *cco1*-promoter is stimulated, *P. putida* pJG-*cco1::gfp* was cultivated in stepwise decreased oxygen saturation, while fluorescence was monitored regularly. In this way, a first increase of fluorescence was detectable below a pO_2_-level of 20% and the signals increased strongly with a further decrease in pO_2_ to 0%. In this context, cellular growth was reduced, but still present for the period tested (Fig. 4, top panel). In contrast, a constant pO_2_ of 40% did not lead to an induction of the biosensor over time, indicating a constantly inactive promoter activity in the presence of high oxygen levels, independent of growth phases (Fig. 4, mid panel). However, a sudden decrease of the pO_2_ from 40 to 0% induced the biosensor and led to slow growth rates of the bacterial culture, yet without a noticeable die-off of the culture, rendering the system suitable for the control of a 2-step rhamnolipid production process, induced by low oxygen levels (Fig. 4, bottom panel).

### Production of rhamnolipids in *P. putida* induced by oxygen limitation

**Fig. 5:**
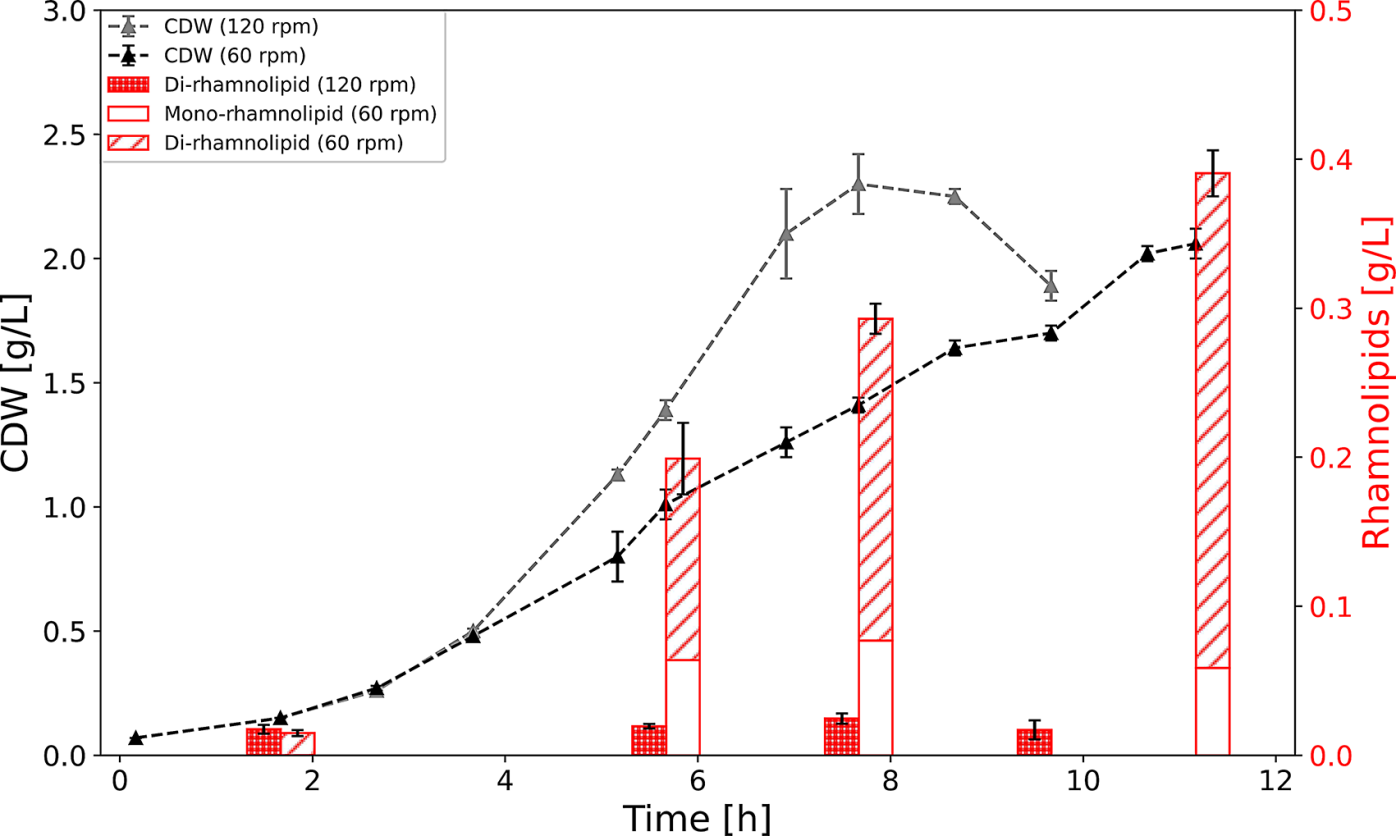
Time course of cell dry weight (CDW) and rhamnolipid production by *P. putida* pJG-*cco1::rhlCAB* under different shake flask agitation conditions. Cultures agitated at 120 rpm (grey triangles) or 60 rpm (black triangles) were monitored for growth (CDW) and rhamnolipid production (red bars)

Overall, the *cco1*-promoter showed a prompt and relatively strong stimulation towards lowered oxygen availability. To apply the promoter for bioprocess control, pJG-*cco1::rhlCAB* was constructed, allowing oxygen-inducible rhamnolipid production in *P. putida*. Reducing shake flask agitation was effective in reducing *P. putida* growth, likely due to reduced oxygen availability, but at very low agitation (40 rpm) also resulted in cell elongation associated with nutrient and oxygen gradients [36]. To induce the oxygen-dependent *rhlCAB* operon by reducing shake flask agitation without non-specific physiological effects, rhamnolipid production was compared at 120 and 60 rpm. As expected, cultures agitated at noninducing 120 rpm produced only negligible amounts of rhamnolipid, while in the cultures agitated at inducing 60 rpm, titers increased to 392 ± 16 mg/L of summed mono- and di-rhamnolipids (Fig. 5).

**Fig. 6:**
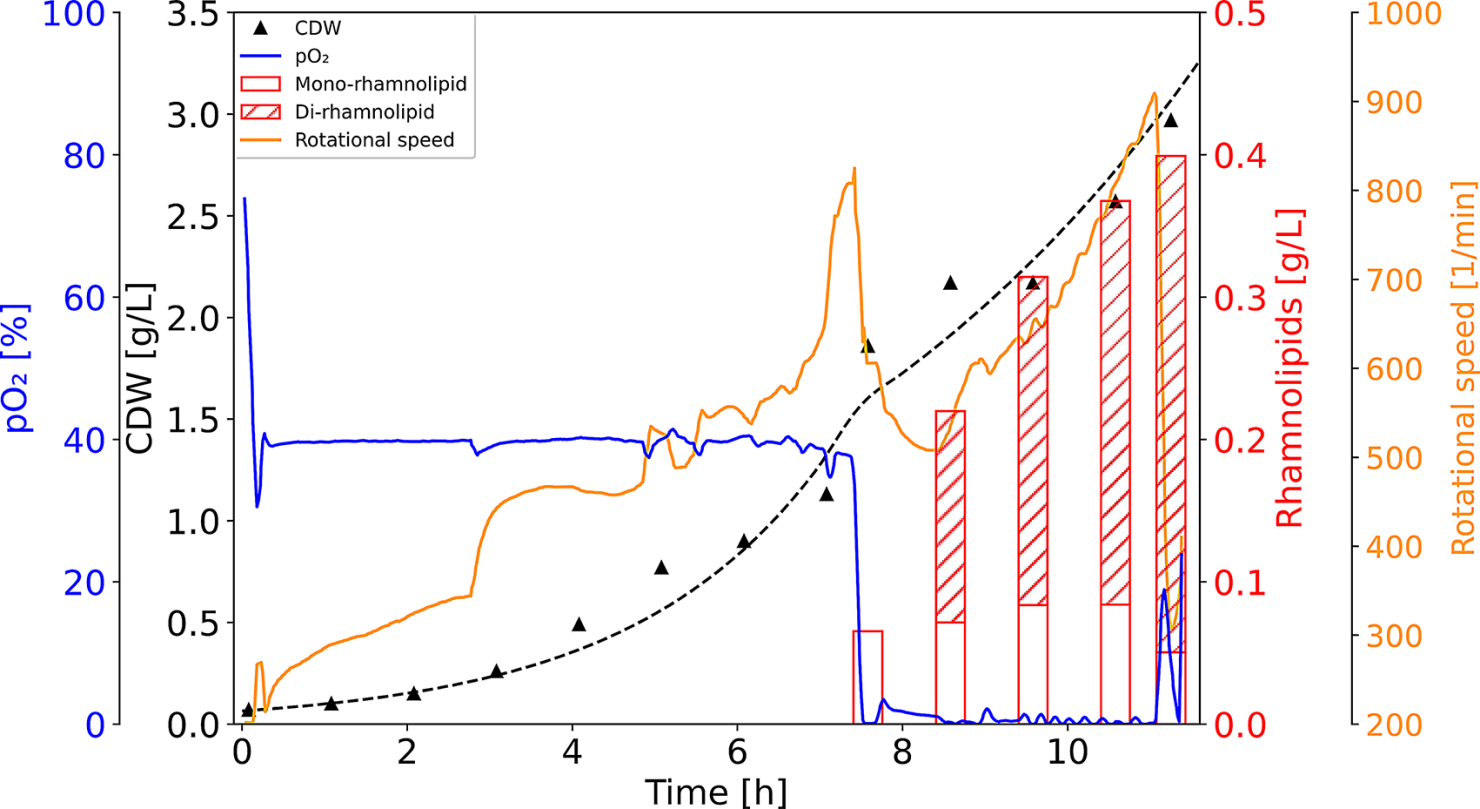
Time course of a dual-phase bioprocess for low-oxygen induced rhamnolipid production. Cell dry weight (CDW, black triangles) of *P. putida* pJG-*cco1::rhlCAB*. The trend of the biomass formation is represented by the dashed line. The rotational speed of the stirrer is displayed in orange. The oxygen availability (pO_2_) is displayed as a blue line, while the red bars indicate the rhamnolipid titers during the fermentation process

Since a sudden drop of pO_2_ did not show a die-off of the bacteria but an induction of the *cco1*-promoter (Fig. 4, bottom panel), *P. putida* pJG-*cco1::rhlCAB* was cultivated in a stirred tank bioreactor in a dual-phase process. In the first phase, high oxygenation levels (40% pO_2_ and 1 L of air per minute) were applied to allow effective biomass formation. At a biomass concentration of approximately 1.5 g_CDW_/L, the second phase was started by setting up microaerobic conditions (0–1% pO_2_ and 0.5 L of air per minute) to induce rhamnolipid production via induction of the transcriptionally fused *cco1*promoter. During the first non-inducing phase, high specific growth rates prevailed (µ = 0.44 h^− 1^) but no rhamnolipid could be detected. During the subsequent microaerobic phase, a reduced cell growth (µ = 0.18 h^− 1^), and production of rhamnolipid was observed with a final titer of approximately 400 mg/L of summed mono- and di-rhamnolipids (Fig. 6).

**Fig. 7:**
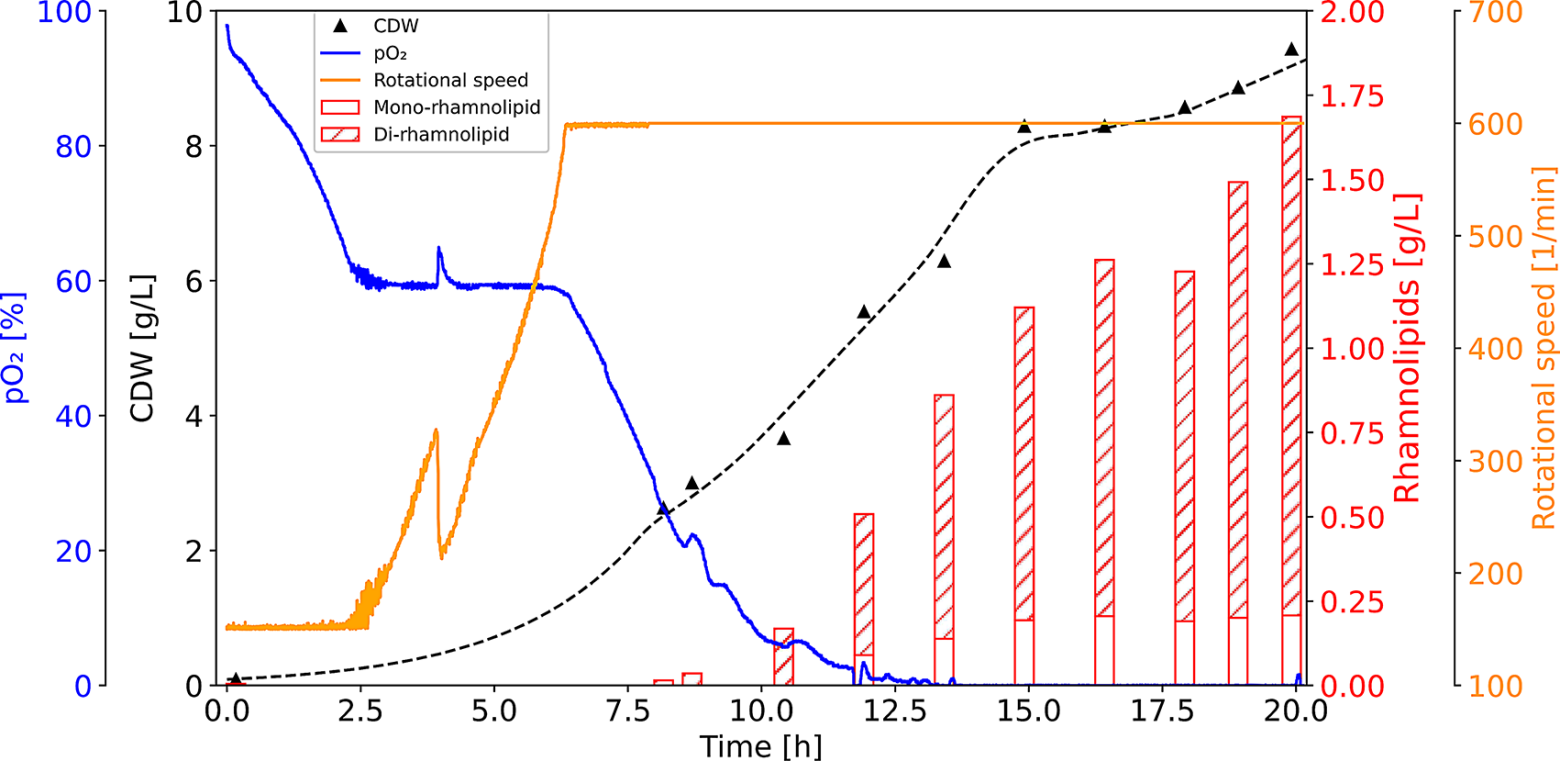
Time-course of an auto-inducing bioprocess for rhamnolipid production. The trend of the cell dry weight (CDW, black triangles) of *P. putida* pJG-*cco1::rhlCAB*. The trend of the biomass formation is represented by the dashed line. The rotational speed is indicated in orange, while the oxygen availability (pO_2_) is shown as a blue line. Red bars indicate rhamnolipid titers produced during the fermentation process

Since the described process control by promoter activation due to low oxygen saturation worked reliably, an auto-inducing rhamnolipid production in a fed-batch process was intended (Fig. 7). For this approach, a bioreactor (15 L working volume) was inoculated with *P. putida* pJG-*cco1::rhlCAB* with a fixed aeration and low maximum stirring rate (limited to 600 rpm) to allow decreasing oxygen saturation during the first hours of the bioreactor cultivation. The oxygen saturation was controlled at not-inducing levels of pO_2_ = 60% in the first phase using the stirrer. Eventually, the maximum stirring rate was reached, and thus, further cell growth led to a decrease of the pO_2_-level over time. After 7.5 h, an exponential feeding was started with a feeding rate of *r* = 0.35 h^− 1^ allowing an extension of the biomass formation. Further cell growth resulted in stimulating conditions with reduced levels of oxygen availability, and when approximately pO_2_ = 20% was reached, the first increase of the rhamnolipid titer was observed, representing an autonomous induction of rhamnolipid production in *P. putida*. At a pO_2_ level of 5%, the feeding rate was lowered to *r* = 0.1 h^− 1^. During the last 7 h of the process, oxygen levels were at pO_2_ = 0%. Notably, these conditions do not reflect anaerobic conditions, since oxygen was supplied constantly via gassing and stirring of the culture. Regarding product formation, rhamnolipid was hardly detectable in the first part of the process (0 to 10 h process time) under high oxygen levels, while high growth rates (µ = 0.42 h^− 1^) were observed. When inducing pO_2_ values of between 10 and 20% were reached, product formation was stimulated and specific growth rates decreased over time, reaching µ = 0.17 h^− 1^ during the second feeding phase. After auto-induction, rhamnolipid titers increased quickly, with a biomass specific product yield of Y_P/X_ = 0.2 g_RL_/g_CDW_ during the low oxygenated period (10 to 15 h process time). Notably, between 15 and 20 h process time, the specific growth rate was only µ = 0.04 h^− 1^, while the product yield in this period was highest with Y_P/X_ = 0.38 g_RL_/g_CDW_. By maintaining low stirring (max. 600 rpm) and aeration rates (0.133 vvm), the experimental setup allowed decoupling of growth and self-induced product formation with a rhamnolipid titer of 1.7 g/L, which is comparable to an aerobic fermentation where 1.5 g/L was achieved, but with a much higher aeration rate of 0.5 vvm [37].

## Discussion

*P. putida* KT2440 has been consistently used in biotechnological applications as a heterologous production host for the production of rhamnolipids as a promising biosurfactant [6]. It is known to be tolerant to microaerobic conditions, and the resilience of *P. putida* towards changing oxygen availability has been described [33]. Hypoxic conditions strongly affect the physiology of the strictly aerobic bacteria, if oxygen demand is greater than the supply [38], leading to molecular adaptations to the low oxygen availability by fine-tuning of their gene expression. The change from oxygen-sufficient to microaerobic conditions should therefore induce genetic responses in *P. putida*, such as the remodeling of the respiratory chain machinery. However, recombinant coupling of the molecular genetic response with rhamnolipid formation in the context of facilitated bioprocess control has not been exploited yet.

To reduce the availability of oxygen in shake flask cultures, several methods have been applied before, such as a variation of filling volume [39] or the reduction of agitation speed [40] to lower the oxygen input into the culture. Both methods can cause additional physiological effects, such as cell elongation, which is probably due to insufficient mixing of the culture, leading to an accumulation of biomass at the bottom of the flask and thus to a local lack of nutrients and oxygen. Cultivation at 40 and 30 rpm resulted in elongated cells (Supplementary Figures S4 and S5). This elongation of cell length has been described before as an adaption of *P. putida* to stressful conditions [36] and proteomic changes in this context have been described [41]. An exchange of the air in the headspace of shake flasks with N_2_ could overcome previously described obstacles, as it allowed a quick exchange of the atmosphere and active oxygen displacement in the medium by mixing. A similar strategy for displacement of oxygen of culture flasks has been applied before in [20]. With this approach, the agitation speed and volume remained unchanged, suggesting the absence of additional non-hypoxic targeted adaptation processes. With this experimental setup, oxygen content could not be measured, however, reduced cell growth suggested limiting oxygen concentrations (Fig. 1). Additionally, untargeted proteome analysis of the oxygen-limited cultures revealed a remodeling of the respiratory chain and an upregulation of stress response proteins (Fig. 2), which confirm oxygen limitation in the cultures.

Regarding the adaptation of *P. putida* to microaerobic conditions, many proteins involved in the respiratory chain were affected by the depletion of oxygen. Exemplarily, the *cbb3*-type cytochrome c oxidases (cco) are unique among the respiration complexes, as these heme copper oxidase (HCO) complexes inherit an extraordinary high affinity to molecular oxygen (the K_M_ value is 6 to 8 times smaller than to other HCOs) and can reduce oxygen very rapidly [32]. In *P. putida*, two copies of this operon, *cco1* (PP_4250 to PP_4253) and *cco2* (PP_4255 to PP_5258), are encoded in adjacent location, however, only *cco1* was detected to be upregulated under hypoxic conditions (Supplementary Table S2). This is in line with previous studies describing strongly increased but rather weak transcription of the *cco1*-operon and *cco2*-operon, respectively, under microaerobic conditions [42]. Notably, the abundance of the protein complex subunits CcoN-I, CcoO-I and CcoP-I increased not solely with advancing time but also decreased with respect to the spatial distance to the joint promoter of the operon, which is located upstream the *ccoN*-I gene (Supplementary Table S2). Upstream of the − 35 region of this promoter, a binding site for ANR is located, which in *P. putida* is homologous to FNR in *E. coli* and a global transcriptional regulator which allows remodeling of the branched respiratory chain of microbes. Under aerobic conditions, ANR is in an inactive monomeric state, while dimerization of ANR during low oxygen availability leads to DNA binding of this transcription factor [43]. At the same time, proteins involved in biosynthesis of heme, cytochromes and ubiquinones are upregulated, which might indicate a general increase of the active components to efficiently exploit traces of oxygen under microaerobic conditions. Several stress related proteins were also present in cells after exposure to oxygen-depleted atmosphere. For instance, the increased abundance of KatA suggests increased occurrence of reactive oxygen species during oxygen limited conditions. The high abundance of Azurin supports this, as this protein was found involved in redox stress in *P. aeruginosa* [44]. Furthermore, under long-term oscillation between high and low oxygen availability, an increased abundance of the short-chain alcohol dehydrogenase AdhP has been described [33]. This enzyme was also upregulated in this study. Also, the carbon storage regulator CsrA, which negatively regulates target gene expression in the stationary phase in *E. coli* [45], was decreased in abundance. At the same time, the ribosome modulation factor (Rmf), which inactivates ribosomes during stationary phase by connecting to 70S ribosomes [46] was more abundant after setting up a N_2_-enriched atmosphere, suggesting reduced translational activity in a low-oxygen and thus low-energy environment. This corresponds with the observation of reduced cell growth after N_2_-mediated oxygen reduction in the headspace of shake flasks (Fig. 1).

The upstream regulatory regions of the stress related coding sequences were of special interest for the construction of oxygen-sensitive strains, as they can be hijacked to express genes of interest, such as the reporter gene *mGFPmut3*, or the rhamnolipid synthesis operon *rhlCAB.* Using the expected promoter regions of three exemplary genes (*cco1*, *adhP* and *rmf*), fluorescent biosensor strains were constructed. To evaluate the functionality of the constructed biosensor strains, shake flask cultivations with decreasing oxygen availability were conducted. Although a reduction of the shaking velocity is unsuitable for investigation of proteomic adaption to oxygen depletion, as it also evokes untargeted responses [41], it can be used to effectively reduce oxygen availability [47], which we expected to be a convenient way to test the functionality of the biosensor strains. In this approach, increased activity after reducing the oxygen supply in shake flask was observed only for the *cco1*-promoter and the *adhP*-promoter. Both strains showed a sudden increase in the biomass-specific fluorescence after decrease of shake flask agitation. The results confirm that untargeted proteome analysis can be used as a method of choice to identify promoter systems stimulated under varying environmental conditions, such as oxygen availability. However, this was not the case for the promoter of the *rmf*-gene (Fig. 3). Although this protein was found in higher abundance after exposure to an oxygen depleted atmosphere (Fig. 2), the respective biosensor did not respond to a decreased oxygen availability by reducing the agitation speed (Fig. 3). Instead, an increase in biosensor-based fluorescence signals could be shown in late stages of cell growth when nutrients became depleted (Supplementary Figure S3). This observation confirms with previous studies describing a ppGpp alarmone-associated increase of the *rmf*-promoter activity in stationary phase in *E. coli* [34, 48]. In *P. putida*, elevated levels of ppGpp were observed during dual limitations of oxygen and glucose [33]. The absence of the biosensor signal after reducing the agitation speed might indicate that the performed reduction of shake flask agitation could not decrease the oxygen-levels far enough to induce the *rmf*-promoter.

Among the three constructed biosensor strains, *P. putida* pJG-*cco1::gfp* was the most promising, since it showed the strongest and most rapid change in fluorescence intensity after reduction of the shaking velocity (Fig. 3). To elucidate a potential pO_2_ range that induces the *cco1*-promoter for subsequent application in rhamnolipid synthesis, *P. putida* pJG-*cco1::gfp* was applied in a series of cultivations with different pO_2_ levels. For this purpose, a stepwise decrease of the pO_2_ from 40 to 0% (4 steps) was performed using a bioreactor system. The results suggested no activity of the *cco1*-promoter in pO_2_ > 20% but increasing activity at pO_2_ ≤ 20% (Fig. 4, top panel). Cultivation for several hours at constant pO_2_ levels confirmed the observations, as the specific fluorescence was constant in cultivations with pO_2_ = 40% (Fig. 4, mid panel), while a steadily increase was detected when the pO_2_ was controlled at 20% (Supplementary Figure S6, upper panel). Interestingly, the activity of the *cco1*-promoter seems to depend on the induction history. When the oxygen level was kept constant at pO_2_ of 20%, the relative fluorescence increased steadily. In subsequent periodically alternating oxygen availability, specific fluorescence increase was very strong, however, when the pO_2_ was controlled at 20% again, a decrease of the specific fluorescence was observed (Supplementary Figure S6, lower panel). This indicates regulation with a pronounced hysteresis of the *cco1*-promoter, since the promoter activity at one oxygen level can assume two different states. A similar behavior was found for an *E. coli* biosensor using the hypoxia-inducible *nar*-promoter which was controlled by the homologous transcription factor FNR [49].

Although the ability of *P. putida* to survive under microaerobic conditions is well-known, this trait so far has not been exploited extensively. However, feasibility of rhamnolipid production under strong oxygen limitation has been confirmed before [17]. Therefore, the use of the well-controllable *cco1-*promoter in combination with the rapid adaption of *P. putida* KT2440 to rapid changes in oxygen availability could be an applicable strategy for rhamnolipid production.

The simultaneous expression of the genes *rhlA*, *rhlB* and *rhlC* in *P. putida* KT2440 leads to the production of a mixture of hydroxyalkanoyloxy alkanoate (HAA), mono- and di-rhamnolipids. An increase of the expression of the *rhlC*-gene, for example by providing an additional promoter, could drive up the proportion of di-rhamnolipids in this mixture [50, 51]. However, to combine the capability to produce a major proportion of di-rhamnolipid and control the gene expression by oxygen availability, the *cco1-*promoter region was transcriptionally coupled to the coding sequences of *rhlA*,* rhlB* and *rhlC*; the latter at the first position of the operon to promote di-rhamnolipid formation, since this should lead to a higher transcription rate of *rhlC*.

For bioprocess control, the almost inactive *cco1*-promoter in high oxygen levels combined with the immediate stimulation of the activity after switch to low oxygen levels, as measured by the biosensor strain, suggested a simple induction of the target gene expression (Fig. 4, bottom panel). Accordingly, this promoter system appears to be suitable for the construction of an oxygen-level controlled rhamnolipid producer strain. Consequently, the initial results showed no production of rhamnolipids in high (120 rpm), and an induction of rhamnolipid synthesis in low (60 rpm) agitation using the di-rhamnolipid production strain *P. putida* pJG-*cco1::rhlCAB* (Fig. 5). The principle of promoter activation for GFP-formation in the biosensor strain *P. putida* pJG-*cco1::gfp* could therefore directly be transferred for the control of the rhamnolipid synthesis using *P. putida* pJG-*cco1::rhlCAB* (Figs. 3 and 5). This production strain allowed the production of a mixture of mono- and di-rhamnolipids, with a major proportion of dirhamnolipids. These observations are consistent with the product composition of the constitutive rhamnolipid production strain *P. putida* pWJ0S described in [50]. Notably, the conditions tested are not necessarily ideal for rhamnolipid production using the *cco1*-promoter, for example, specific productivity could be higher at lower agitation. In this context, future studies should aim for finding an ideal tradeoff between *cco1*-promoter activation and metabolic activity in reduced oxygen availability.

The process transfer from shake flask to 6 L bioreactor-scale resulted in a clear separation of growth and production phase, when the pO_2_ was first controlled at high levels (pO_2_ = 40%) and subsequently was dropped to microaerobic conditions (pO_2_ = 0–1%) by reduction of the stirrer speed (Fig. 6), which reduces the foam height and stability in stirred tank reactors [11]. Notably, overfoaming of the bioreactor could be prevented by this stirrer speed reduction without the addition of antifoaming agent. Interestingly, during cultivation of *P. putida*, the sudden decrease of the oxygen availability did not lead to a lag-phase or a decrease in cell density, as for example has been described for the industrial workhorse *Bacillus subtilis.* In this context, a pO_2_-drop from 20 to 0% led to an almost complete loss of biomass, and a slow and stepwise decrease of the pO_2_-level was necessary to overcome this obstacle [19]. The stable growth of *P. putida* after a rapid pO_2_-drop from 40 to 0% (Fig. 6) and during rapidly alternating oxygen availability between 20 and 0% (Supplementary Figure S6, bottom panel) underlines its robustness and potential as production host for biosurfactants in industrial biotechnology. The control of oxygen in fermentation makes the *cco1*-promoter an attractive candidate for industrial applications that require separation of growth and production phases without the need for external inducers. Therefore, the *cco1*-promoter-driven expression system could be applied not only for rhamnolipid production but also for the biosynthesis of other bioproducts in *P. putida*. By modulation of the promoter sequence, promoter strength can be increased [52]. In this context, future studies should aim to increase the strength of the *cco1*-promoter, as it is well-controllable but probably rather weak, e.g. compared to the SynPro8-promoter [13], which was specifically designed as a strong, constitutive promoter.

In a next step, the oxygen level-induced rhamnolipid production was successfully applied to establish an autonomously inducing rhamnolipid bioproduction process (Fig. 7). For this approach, a fed-batch bioprocess was conducted, in which parameters for aeration were kept constant and stirring rates were limited to low maximum rates. Since the oxygen consumption increased in growing biomass, a limitation in these parameters led automatically to low oxygen values over time, which resulted in a stimulation of the *cco1*-promoter and *rhlCAB* operon with an associated rhamnolipid production in *P. putida* pJG-*cco1::rhlCAB* (approximately when pO_2_ ≤ 20%). In this way, a decrease of the bacterial growth rate was initiated while the rhamnolipid production phase was stimulated. During the production phase, the main sources of foam formation were limited, requiring little monitoring of the bioreactor. Foam formation is difficult to quantify, however, measurement of head space filled with foam in different aeration and stirring rates can give an impression of how keeping these parameters low prevents foam formation (Supplementary Figure S7).

By coupling the rhamnolipid production to the prevailing oxygen level, the process could be sharply divided into a growth phase without quantitative rhamnolipid production and a production phase with very low growth rates, but relatively high biomass-specific product yield of up to Y_P/X_ = 0.38 g_RL_/g_CDW_. The integrated design of the process, covering minimal fermenter regulation and the use of the tightly regulated promoter of the *ccb3*-type cytochrome oxidase operon allowed demonstrating how self-inducing bioprocesses can be implemented also in difficult-to-handle bioprocesses.

## Conclusions

Foam formation often is a main obstacle for efficient production of biosurfactants such as rhamnolipids. Microaerobic fermentation is a promising but underexplored strategy for bioprocess control, as low aeration and stirring rates can effectively reduce foam formation in bioreactors. In addition, low oxygen availability leads to microbial adaptation, which can be used to induce biosynthetic genes. The robust model bacterium *P. putida* KT2440 can adapt rapidly to limited oxygen availability by remodeling its branched respiratory chain and expression of oxygen-affine cytochrome c oxidases. The utilization of the promoter of the *cbb3*-type cytochrome c oxidase operon allowed the design of easily controllable rhamnolipid production processes. A threshold of 20% oxygen saturation was found to induce this promoter system. By limiting the aeration and stirring rates of the bioreactor, sources of foam formation were effectively reduced, and an oxygen dependent auto-inducing fed-batch process could be implemented, which represents a novel strategy to produce surface-active compounds. The use of oxygen-controlled promoters like the *cco1*-promoter also holds promise for other biotechnological applications beyond rhamnolipid production. This system could potentially be applied to the controlled production of other foaming compounds, where a distinct separation between growth and production phases is beneficial. Moreover, this approach aligns well with the growing demand for sustainable and efficient bioprocesses, reducing the need for costly chemical inducers and simplifying downstream processing.

## Electronic supplementary material

Below is the link to the electronic supplementary material.

Supplementary Material 1

Supplementary Material 2

## Data Availability

No datasets were generated or analysed during the current study.
