# Supplementary material for: Bioprocess exploitation of microaerobic auto-induction using the example of rhamnolipid biosynthesis in Pseudomonas putida KT2440
Source: J Biol Eng. 2025 Jan 18;19:8. doi: 10.1186/s13036-025-00478-z (PMC11742490; doi:10.1186/s13036-025-00478-z)
Supplement: Supplementary file 2 — Supplementary Material 2 [file 13036_2025_478_MOESM2_ESM.pdf]

Supplementary Data

Bioprocess exploitation of microaerobic auto-induction using the example of rhamnolipid biosynthesis in *Pseudomonas putida* KT2440.

Jakob Grether[1] (jakob.grether@uni-hohenheim.de), Holger Dittmann[1] (holger.dittmann@uni-hohenheim.de), Leon Willems[1] (leon.willems98@googlemail.com), Tabea Schmiegelt[1] (t.schmiegelt@freenet.de), Elvio Henrique Benatto Perino[1] (eperino@uni-hohenheim.de), Philipp Hubel[2] (philipp.hubel@uni-hohenheim.de), Lars Lilge[1]* (lars.lilge@uni-hohenheim.de), Rudolf Hausmann[1] (rudolf.hausmann@uni-hohenheim.de)

[1]Department of Bioprocess Engineering, Institute of Food Science and Biotechnology, University of Hohenheim, Fruwirthstr. 12, 70599 Stuttgart, Germany.

[2]Core Facility Hohenheim, Mass Spectrometry Core Facility, University of Hohenheim, Ottilie-Zeller-Weg 2, 70599 Stuttgart, Germany.

* corresponding author: Lars Lilge, lars.lilge@uni-hohenheim.de

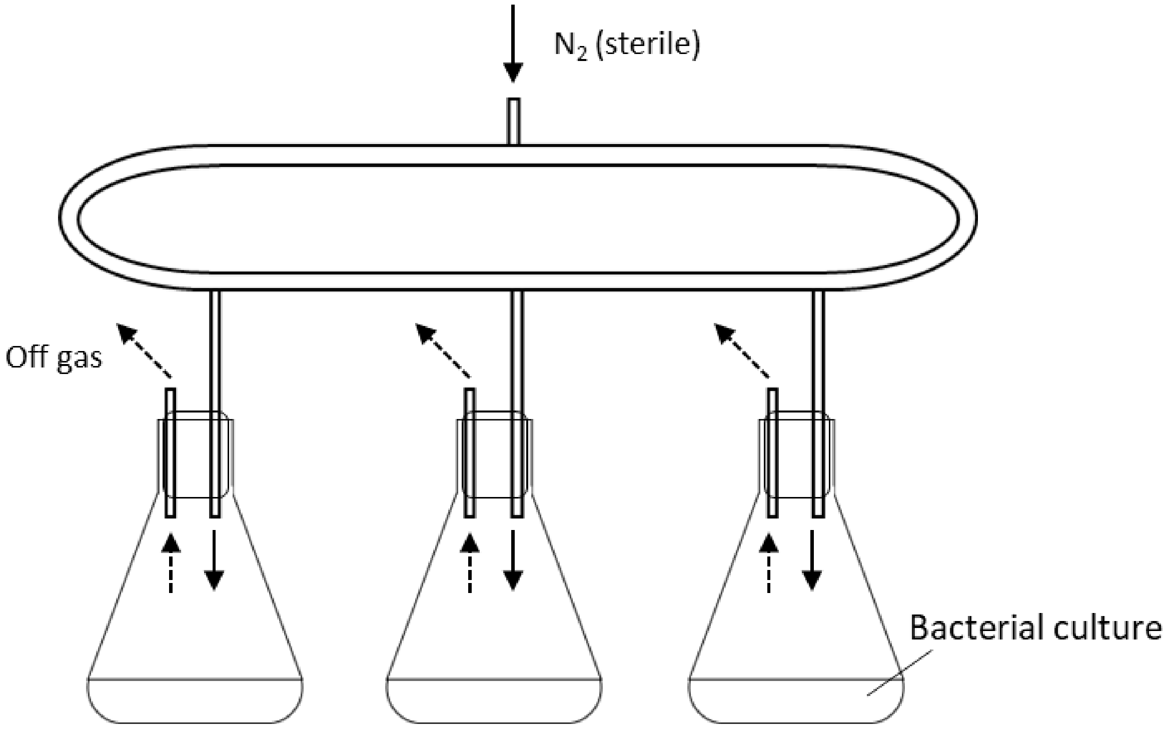

Figure S1: Experimental setup for atmosphere exchange in shake flasks. Three shake flasks were connected via silicone tubes. Tubes were routed through the shake flask plugs, allowing the inflow of sterile nitrogen gas (N_2) into the flask headspace (arrow), which leads to an outflow of the air (dashed arrow). Tubes were clamped after atmosphere exchange, and plugs were air tightened to prevent loss of N_2 in the shake flask. Samples was enabled without opening the flasks via long cannulas reaching into the bacterial culture.

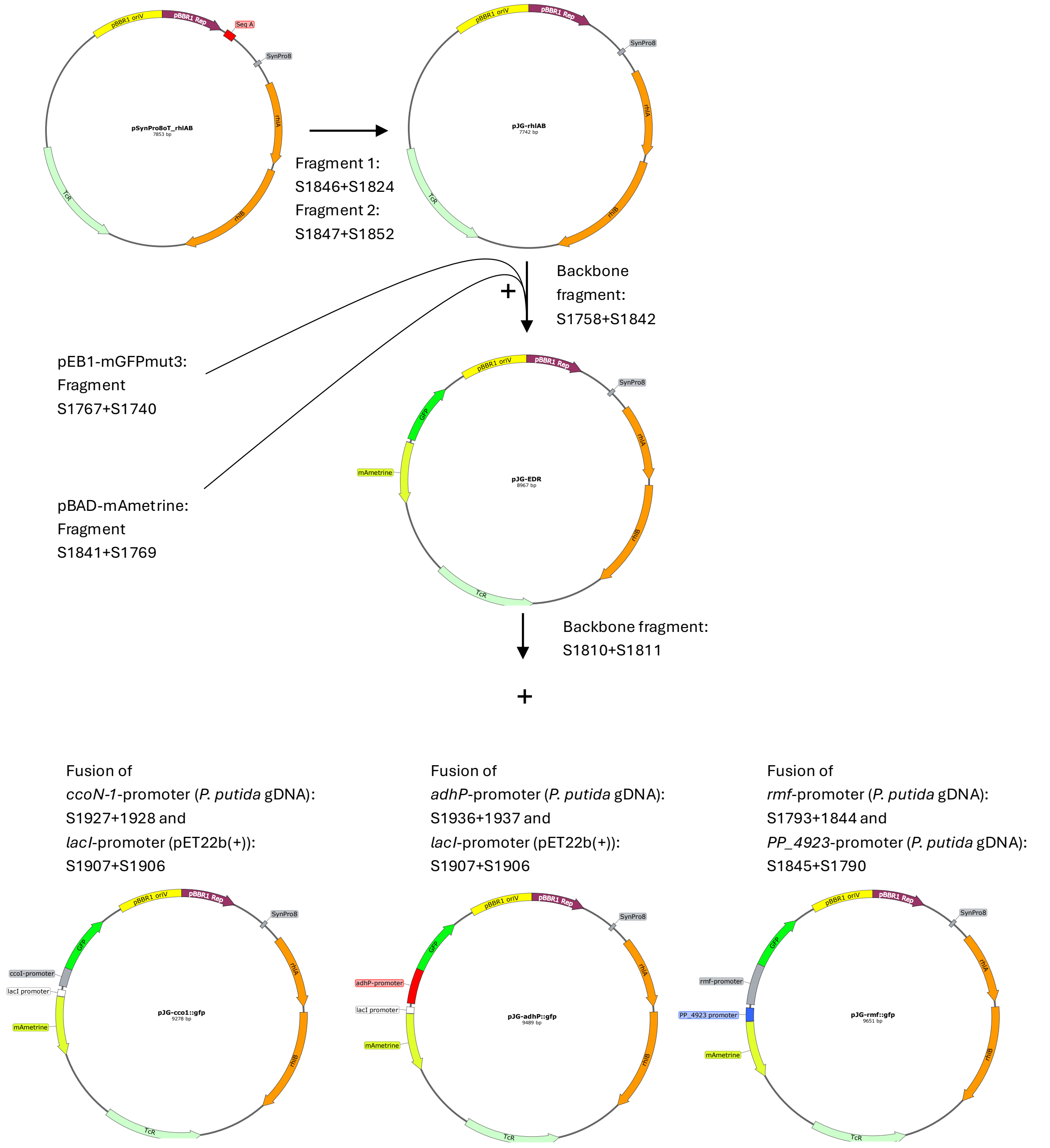

Figure S2: Schematic cloning strategy of the biosensor plasmids. In the first step, a non-functional region (here referred to as Seq A) in pSynPro8oT_rhlAB was deleted by amplification and subsequent Gibson Assembly of the backbone fragments 1 and 2, leading to pJG-rhlAB. To generate pJG-EDR, which served as a parental plasmid for the generation of the biosensor plasmids, the backbone of pJG-rhlAB was amplified and combined via Gibson Assembly with the coding regions of *mGFPmut3* and *mAmetrine*, which were amplified from pEB1-mGFPmut3 and pBAD-mAmetrine, respectively. The backbone of pJG-EDR was then assembled with fusions of upstream regions of target genes, which are simplified here as promoters. In detail, for pJG-cco1::gfp and pJG-adhP::gfp, the promoter of *ccoN-1* and adhP, respectively, was fused with the *lacI*-promoter. For pJG-rmf::gfp, the promoter of *rmf* was fused with the promoter of *PP_4923*. While the expression of *mAmetrine* under control of the *lacI* or *PP_4923* promoter is no topic of this manuscript, the *mGFPmut3*-gene of each biosensor plasmid was in transcriptional fusion with the *ccoN-1*, *adhP*, or *rmf* promoter, respectively, allowing GFP-expression under inductive conditions. SynPro8, synthetic constitutive promoter for expression of *rhlA* and *rhlB*; *rhlA*, 3-(3-hydroxydecanoyloxy)decanoate synthase; *rhlB*, rhamnosyltransferase 1; *TcR*, tetracycline resistance gene; *pBBR1 oriV*, origin of replication; *pBBR1 Rep*, replication protein; *GFP*, coding sequence of mGFPmut3; *mAmetrine*, coding sequence of mAmetrine

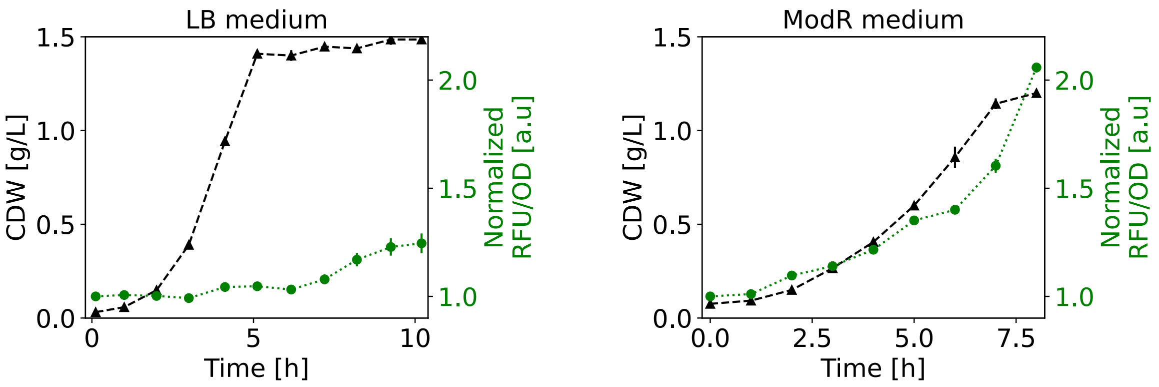

Figure S3: Time course of cell dry weight (CDW, black triangles) and specific fluorescence (RFU/OD, green dots) during cultivation of the biosensor strain P. putida pJG-*rmf::gfp* in LB (left) and ModR (right) medium. In LB medium, specific fluorescence was constant over approximately 6 hours. After transitioning to stationary phase, an increase of the specific fluorescence was observed. In ModR medium (2.5 g/L glucose as sole carbon source) a steady increase of the fluorescence was observed, which intensified during transitioning to stationary phase.

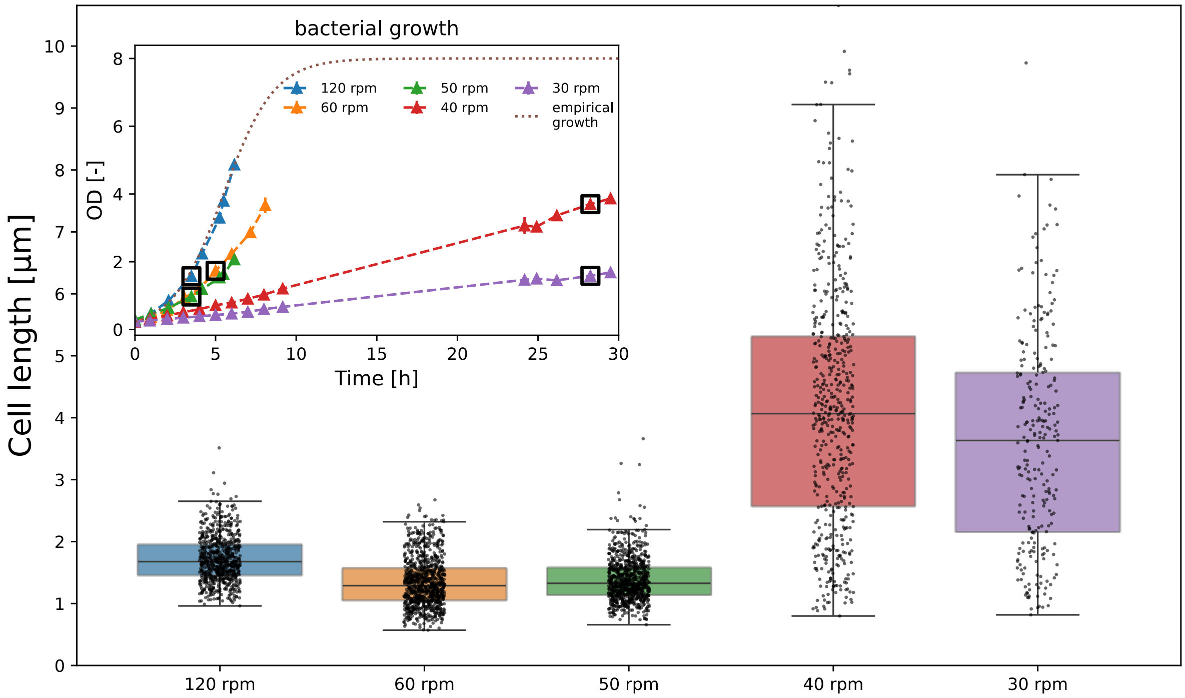

Figure S4: Cell length (Box plots) and time course of growth of P. putida pSynPro8oT_rhlAB in different shake flask agitation (rounds per minute, rpm) as indicated. Bacteria were cultivated in ModR medium (5 g/L glucose as sole carbon source). In high agitation (120 rpm) bacterial cultures grew exponentially, and optical density (OD) empirically reaches final value of around 8.0 (dotted line). With decreasing agitation, growth rates decrease, and almost linear-like growth curves are observed in 40 and 30 rpm. Under these conditions, cell lengths strongly increase over time, as indicated with the box plots. Every dot represents a single bacterial cell. Sampling for microscopy was done in the mid growth phase, as indicated by the black squares.

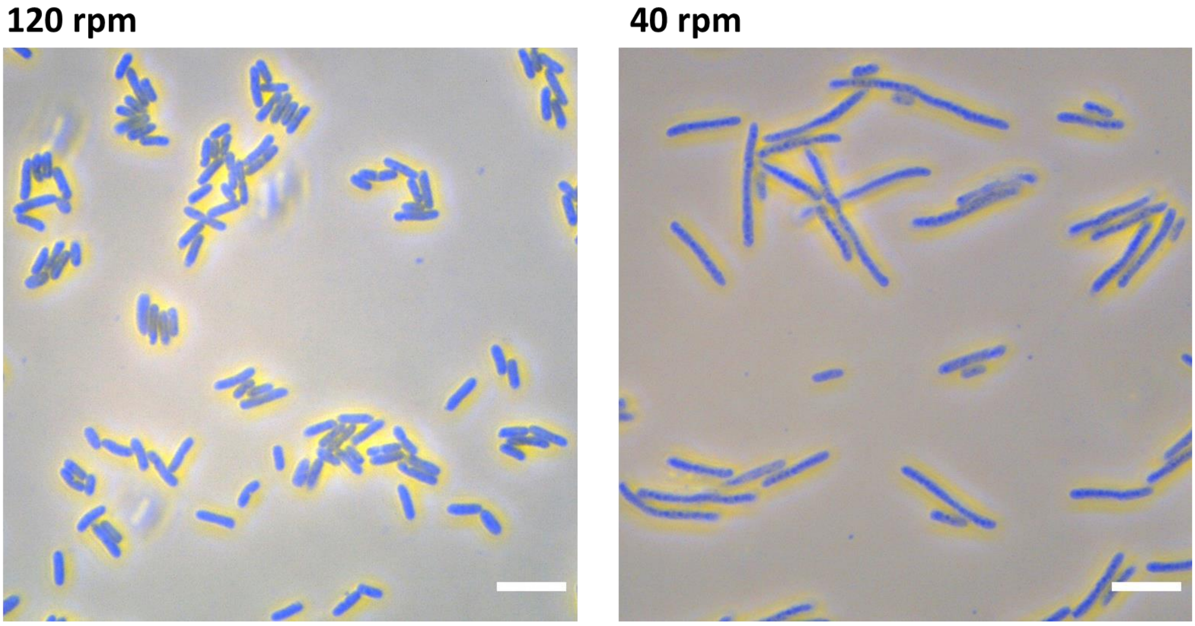

Figure S5: Representative photograph of P. putida cells, cultivated under high (120 rpm) and low (40 rpm) agitation. Under low agitation, cell length increases strongly, as described in Figures S4. White bars represent 5 µm.

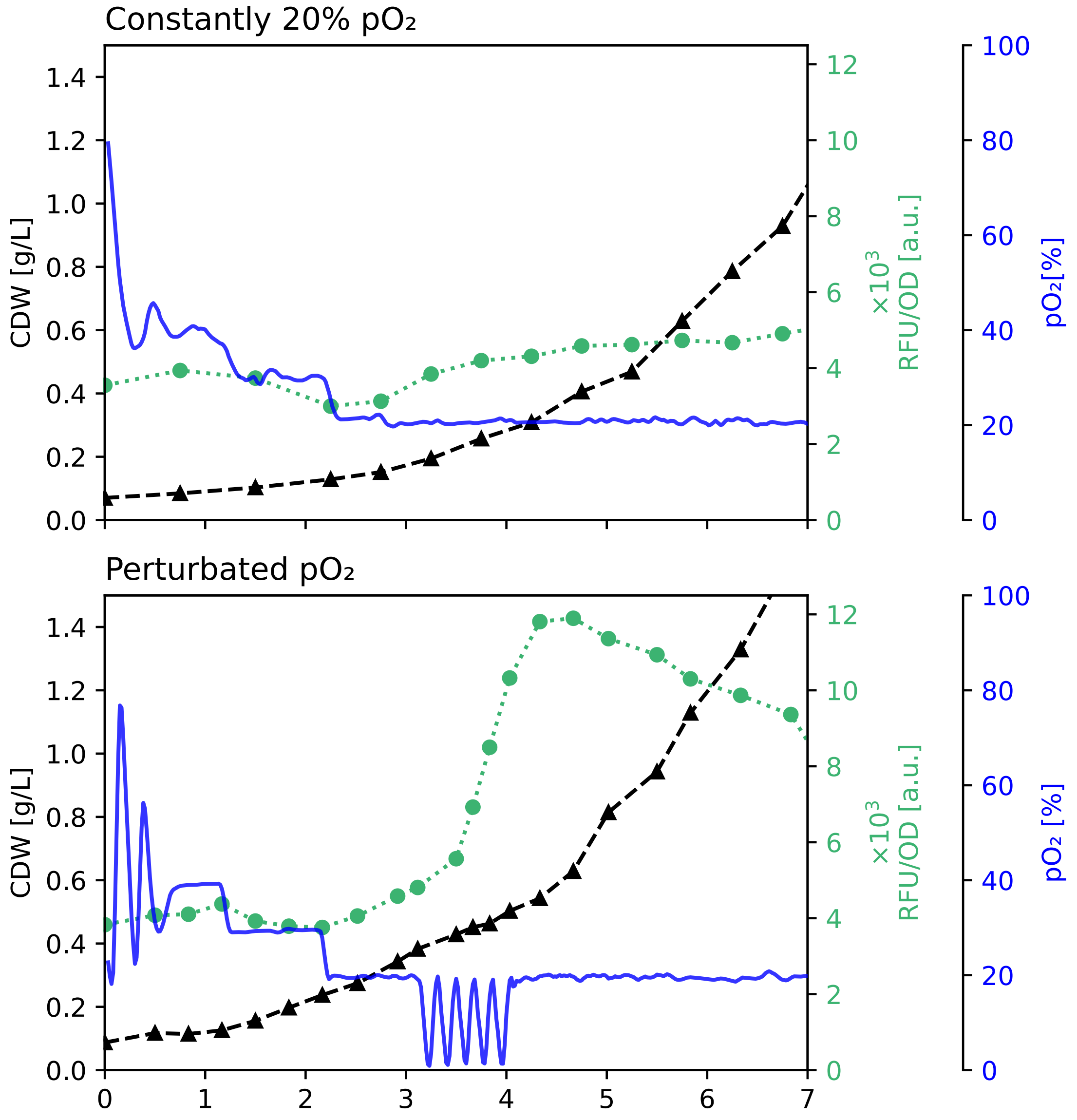

Figure S6: Time course of cell dry weight (CDW, black triangles) and specific fluorescence (RFU/OD, green dots) under different oxygen availability (pO2, blue line). In this experiments, the oxygen sensitive biosensor strain P. putida pJG-*cco1::gfp* was used. Upper panel: Oxygen availability was decreased stepwise to 20% and then held constant at this level. A slight increase of specific fluorescence was observed under these conditions. Lower panel: Oxygen availability was stepwise decreased to 20%, and after 1 hour at this oxygen level, perturbations with changing pO2-levels were applied. During these perturbations, specific fluorescence strongly increased. After that, the pO2 was controlled at 20% again, which now led to decreasing specific fluorescence.

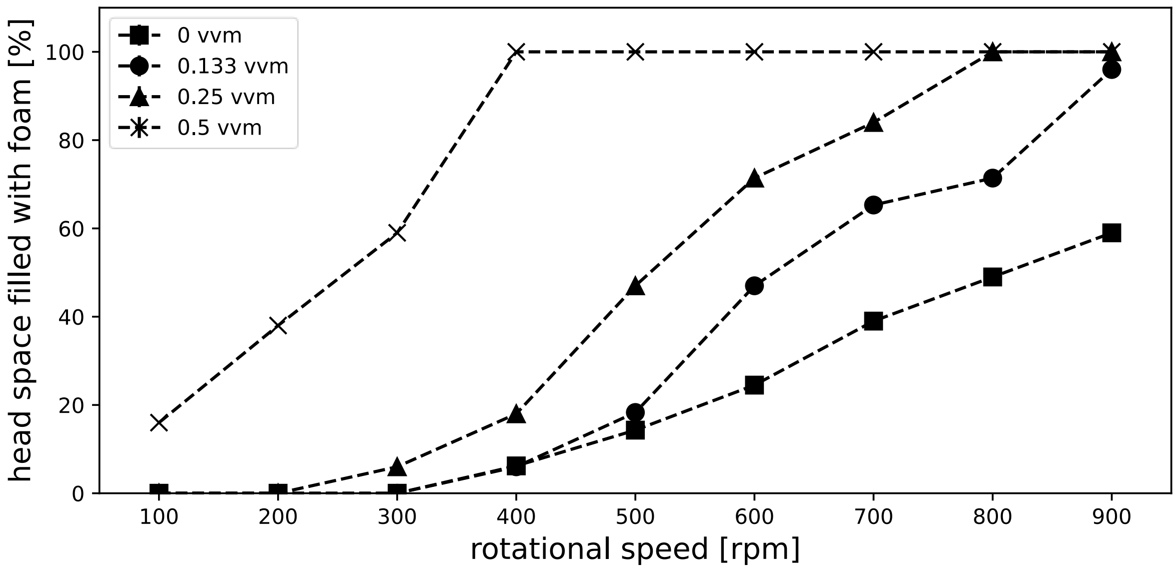

Figure S7: Representation of foam in bioreactor in different combinations of aeration and stirring rates. A 10L stirred tank bioreactor system equipped with two 6-blade turbines was filled with 6 L of ModR medium containing 1.7 g/L rhamnolipid. The system was aerated at different rates (0 vvm, 0.133 vvm, 0.25 vvm and 0.5 vvm) and stirred with varying rotational speed (100 to 900 rpm). The head space filled with foam was measured for every combination as indicated. Foam formation was lower when aeration and stirring rates were low.

Table S1 Primers and DNA templates used for the cloning procedure.

Primer	Sequence (5'-3')	Template (function)	Product
S1846	actatcggctgtaggtttttatcaggctctgg	pSynPro8oT_rhlAB [16] (construction of plasmid pJG-rhlAB)	pSynPro8oT_rhlAB fragment 1
S1824	cacttatgactgtcttct		
S1847	gagcctgataaaaacctacagccgatagtctgg	pSynPro8oT_rhlAB (construction of plasmid pJG-rhlAB)	pSynPro8oT_rhlAB fragment 2
S1852	ctaggtattatgctagccggaagagagtcaattcagg		
S1758	gaacgctctcctgagtaggacaaattactcactagactttgcttcg	pJG-rhlAB (construction of biosensor plasmid pJG-EDR)	Backbone of pJG-rhlAB
S1842	gccacaagggcgctttagtttgttttgcattcaggcgttctacg		
S1841	aacaaactaaagcgcccttgtggcgctttagttttatgcctggcagttccctac	pBad-mAmetrine [15]	CDS of *mAmetrine*
S1769	ctcgtgcgttcaacaccattaggcagggtaccatggtgagcaagggcgag		
S1767	accctgcctaatggtgttgaacgcacgagctcatgagtaaaggagaagaacttttcac	pEB1-mGFPmut3 [14]	CDS of *mGFPmut3* [17]
S1740	gaagcaaagtctagtgagtaatttgtcctactcaggagagcg		
S1810	atgagtaaaggagaagaacttttcactgga	pJG-EDR (parental plasmid for biosensor plasmids)	Backbone of pJG-EDR
S1811	atggtgagcaagggcgag		
S1907	aaagcctccggtcggaggcttttgactggtaccgacaccatcgaatgg	pET22b(+) (Novagen®)	*lacI*-promoter
S1906	gaacagctcctcgcccttgctcaccatattcaccaccctgaattg		
S1927	aaagcctccgaccggaggcttttgactgagctccatttcgtccatgaacc	*P. putida* KT2440 gDNA	224 bp upstream region of *ccoN-1* (*PP_4250*)
S1928	ccagtgaaaagttcttctcctttactcatgtatgggttcccat		
S1936	aaagcctccgaccggaggcttttgactgagctcgctggctcctgggataatg	*P. putida* KT2440 gDNA	435 bp upstream region of *adhP* (*PP_3839*)
S1937	ccagtgaaaagttcttctcctttactcatgagagcctccgtgtc		
S1793	gtgaaaagttcttctcctttactcatagggtgttgccctca	*P. putida* KT2440 gDNA	510 bp upstream region of *rmf* (*PP_5502*)
S1844	aaagcctccgaccggaggcttttgactgagctccgatgtttgcccgtacac		
S1845	aaagcctccggtcggaggcttttgactggtaccaacatgcgcagtctagact	*P. putida* KT2440 gDNA	165 bp upstream region of *PP_4923*
S1790	ctcctcgcccttgctcaccatagaggcaatccttgtacgaa		
For pJG-cco1::rhlCAB			
BB1	ggttcatggacgaaatggagctcacgcctggtgctacgc	pJG-rhlAB	Backbone of pJG-rhlAB
BB2	aaggccaaggcctagcgtggcagcgatagctgtttgcc		

Primer	Sequence (5'-3')	Template (function)	Product
ccoi1	ttcaggcgtagcaccaggcgtgagctccat ttcgtccatgaacc	*P. putida* KT2440 gDNA	Upstream region of *ccoN-I* (*PP_4250*) for pJG-cco1::rhlCAB
ccoi2	ccatgtctatccggtccatgtatgggttccc atccacg		
rhlC1	taaccgtggatgggaacccatacatggacc ggatagacatggg	*P. aeruginosa* gDNA (DSM 1988)	CDS of *rhlC* (PA1130)
rhlC2	aaacagctatcgctgccacgctaggccttg gccttgc		
